# Effectiveness of *In Virtuo* Exposure and Response Prevention Treatment Using Cognitive–Behavioral Therapy for Obsessive–Compulsive Disorder: A Study Based on a Single-Case Study Protocol

**DOI:** 10.3389/fpsyt.2016.00099

**Published:** 2016-06-13

**Authors:** Mylène Laforest, Stéphane Bouchard, Jessie Bossé, Olivier Mesly

**Affiliations:** ^1^University of Ottawa, Ottawa, ON, Canada; ^2^Université du Québec en Outaouais, Gatineau, QC, Canada; ^3^Université Sainte-Anne, Pointe-de-l’Église, NS, Canada

**Keywords:** virtual reality, obsessive–compulsive disorder, CBT, response prevention, exposure

## Abstract

Obsessive–compulsive disorder (OCD) is characterized by the presence of distressing, recurrent and intrusive thoughts, impulses, or doubts as well as behavioral or mental rituals. OCD has various subtypes, including the fear of contamination in which individuals fear bacteria, germs, disease, or bodily secretions, and engage in clinically significant cleaning and avoidance rituals. Cognitive–behavioral therapy (CBT) is an effective treatment for OCD and involves, among other therapeutic strategies, exposing patients to feared stimuli while preventing them to engage in compulsive behaviors. In recent years, virtual reality (VR) has shown the potential of *in virtuo* exposure with people suffering from anxiety disorders and OCD. The objective of this pilot study is to examine the effectiveness of a CBT program where exposure in conducted *in virtuo*. Three adults suffering from OCD with a dominant subtype of contamination were enrolled in a single-case design with multiple baselines across participants. The presence and intensity of obsessions and compulsions were assessed daily during baselines of 3-, 4-, or 5-week, and a 12-session treatment. Follow-up information was gathered after 4 and 8 months. Treatment outcome is assessed with visual inspection of the graphs and ARMA time-series analyses. Clinical information, self-reports, and details of the treatment are provided for each patient. Statistical analyses for the time-series data revealed a statistically significant improvement in all three participants, but global improvement is considered positive for only two. This study innovates in proving preliminary support for the usefulness of VR in the CBT of OCD with contamination features.

## Introduction

Obsessive–compulsive disorder (OCD) is a severe and debilitating mental disorder characterized by recurrent and intrusive thoughts, impulses, or doubts that cause marked anxiety in individual, as well as behavioral or mental rituals that are performed in order to reduce distress caused by the obsessions. Ninety percent of the general population shares similar concerns as people diagnosed with OCD (1). The difference between the two populations is the importance given to the presence of thoughts; people with OCD attribute much more importance to their thoughts and their ability to control them than the general population. Despite the fact that most people suffering from OCD are aware of the irrational aspect of their thoughts, they are unable to stop the unwanted thoughts from reoccurring. Compulsions or rituals therefore develop in an effort to control thoughts and bring temporary relief from their anxiety.

In terms of obsessions, fears may be related to contamination, doubts, orderliness, religion, morality, aggression, or sexuality. When it comes to compulsions, the most common are cleaning/washing, checking, ordering/symmetry, and accumulating. More precisely, people with a fear of contamination are absorbed by worries such as fear of microbes, bacteria, diseases, bodily fluids, and chemicals. To ease their fears, they feel the urge to wash excessively (hands, body, teeth, clothes), clean household items or personal property, and avoid situations in which there are risks of contamination. OCD symptoms and avoidance behaviors are heterogeneous and led to the development of various subtypes (2), such as patients predominantly concerned by contamination and washing, checking and verifying, or hoarding. Subtyping OCD symptoms does not necessarily imply the need to develop different theoretical models and treatments for each subtype (2, 3), but is very important in terms of clarifying the stimuli that will be targeted in psychotherapy.

Various theoretical models explaining the occurrence of OCD have been proposed, referring in particular to various factors including biological (4, 5), genetic (6), and psychological (1, 7–9). In relation to these theoretical models, most researchers agree that OCD develops and evolves because of both cognitive and behavioral factors. On the one hand, thoughts are interpreted as catastrophic. Imminent danger is associated with thoughts stemming from dysfunctional associations with threat and danger. These associations with perceived threat can be linked to an inflated sense of responsibility, over importance given to thoughts or the need to control them, overestimation of danger, intolerance of uncertainty, perfectionism, or pathological doubt (1, 10, 11).

On the other hand, behavioral factors are also present in the development of OCD since individuals try to relieve distress by accomplishing rituals or avoidance behaviors, which have short-term effects in reducing suffering. By resorting to compulsions, people with OCD fall into an avoidance trap; that is, avoidance behaviors prevent the possibility of confronting fears in order to learn that there is no danger and that they can cope with the stimuli and tolerate discomfort. In other words, by avoiding at all costs to confront or to tolerate feared situations without resorting to compulsions, individuals remain convinced that their compulsions prevent feared consequences. Fear can thereafter extend and generalize to other objects or situations, leading to further compulsions.

Cognitive–behavioral therapy (CBT) is an effective treatment for OCD, especially due to its technique of exposure and response prevention (12, 13). This treatment involves several components (e.g., case formulation, psychoeducation, cognitive restructuring, and relapse prevention), including repeated and prolonged exposure to stimuli and situations causing distress. Exposure usually takes place gradually, in collaboration with the patient who is invited to confront moderate fears at the beginning and progressively move on to fears triggering higher levels of anxiety. During or after exposures, the participant is asked to refrain from performing rituals by tolerating anxiety, despite the strong urge to resort to compulsions. Up until now, exposure and response prevention have been conducted mostly in reality (*in vivo*) or in imagination. For example, people suffering from OCD with contamination subtype are exposed to contaminated stimuli (i.e., going into a public restroom) and then asked to tolerate anxiety without resorting to washing rituals. However, research suggests that traditional exposure may have some limitations (i.e., participants may find it difficult to imagine situations and therapists may struggle in gaging the intensity of the anxiety-provoking stimuli). Despite evidence demonstrating its effectiveness in the treatment of OCD, some clinicians are reluctant to use *in vivo* exposure because they feel less knowledgeable or lack training as well as equipment for its application (14). Finally, participants often express strong apprehension about conducting exposure in a real situation (15).

Using virtual reality (VR) in therapy is a new approach that has been introduced in order to address some inherent limitations of traditional exposure techniques involved in the treatment of anxiety disorders. VR is defined as an application that allows users to navigate and interact in real time with a three-dimensional environment generated by computers (16) and can be used to conduct exposure and response prevention. *In virtuo* exposure allows user to be exposed to anxiety-provoking stimuli (while following the same principles as *in vivo* exposure), but relies on computer-generated situations. Recent studies have demonstrated the effectiveness of VR in the treatment of various anxiety disorders (13, 17–21). VR offers several advantages for exposure (21), such as easy access to stimuli, increased control over exposure stimuli, reassuring and flexible context of exposure, bypassing problems associated with mental imagery, and make exposure more enticing to for patients.

Only a few studies have been published on VR and OCD, these having focused on the checking and verification subtype [for a review, see Ref. (22)]. Results showed that people suffering from OCD reported significantly higher anxiety levels compared with the control group (23), which suggest that VR can be used as a tool for inducing anxiety in this clinical population and consequently supports its usefulness as a medium for exposure as part of CBT. VR can also be used a behavioral measure of verification with OCD patients (24).

To the best of our knowledge, there is no treatment study focusing on the contribution of VR in CBT for OCD with the contamination subtype. Laforest and Bouchard (25) (in revision) validated the potential of immersions in VR for conducting *in virtuo* exposure with participants suffering from OCD with a predominant subtype of contamination. They compared subjective and physiological reactions of a control group of 20 non-OCD adults to a group of 12 adults suffering from OCD with contamination subtype when immersed in a “neutral” VR environment and a “contaminated” public toilet. Results showed that people suffering from OCD reported a significantly higher level of anxiety during the immersion in the contaminated virtual environment when compared with the control group on both state anxiety and heart rate. Having shown the effectiveness of VR to elicit anxiety when confronted with stimuli relevant for the treatment of OCD, the next step is to evaluate its potential as a tool used to conduct *in virtuo* exposure and response prevention.

The main objective of the current study was to examine the efficacy of a therapeutic treatment using VR for the contamination subtype of OCD. Four variables were examined: (a) the presence of obsessions, (b) the presence of compulsions, (c) the intensity of obsessions, and (d) the intensity of compulsions. The hypotheses were that presence and intensity of obsessions and compulsions would decrease following the introduction of CBT using *in virtuo* exposure.

## Materials and Methods

This study uses an experimental protocol with multiple baselines across participants (26–28) in order to test the effectiveness of a VR treatment in reducing OCD symptoms. In order to demonstrate the effectiveness of the intervention, participants’ self-reported symptoms are expected to be “stable” (i.e., not in a decreasing trend) at baseline level and decrease when treatment was introduced. This experimental design allows for maximization of internal validity by introducing treatment at different points in time for different individuals and provides experimental control over the possible effects of maturation, historical factors, and the impact of life events (26–28) by demonstrating that each participant improves when treatment is introduced. Participants were *a priori* randomly assigned to a baseline of 3, 4, or 5 weeks of “stability” before starting treatment. They were informed of the protocol being used and how they had to self-monitor the presence and intensity of their obsessions and compulsions in a daily diary during the duration of the entire study. After the baseline period, participants received a manualized cognitive–behavioral therapy with VR exposure during a 12-week period [based on Foa and Kozak (29)]. Pre- and post-questionnaires were added to the protocol in order to obtain additional descriptive data in regard to the therapeutic progress of each participant.

### Participants

There are no rules specifying the required number of participants in multiple baselines protocols, but several case studies have been carried out with three or four participants (7, 8, 30, 31). Participants were recruited from ads in university and local newspapers inviting men and women between the ages of 18 and 65 years suffering from OCD with a primary subtype of contamination to contact the researchers. All participants were involved in a previous validation study of the VR environment [Ref. (25), in revision].

The following exclusion criteria were set *a priori*: (a) a main diagnosis other than OCD; (b) a secondary diagnosis of schizophrenia, bipolar disorder, organic brain disorder, intellectual disability, substance abuse or dependence, or suicidal ideations; (c) a primary subtype of OCD other than one related to obsessions of contamination and cleaning compulsions; (d) the presence of a physical condition contraindicating participation in the study (i.e., epilepsy, visual disturbances); (e) a duration of OCD of <12 months. Additionally, participants who had been taking anxiolytic medication to treat their OCD had to be symptomatic and pharmacotherapy must have started at least 6 months prior in order to ensure the effectiveness of the dosage. The dosage and medication type could not change following entry in the study. Finally, participants could not undergo parallel treatments, whether pharmacological or psychological, other than the one being offered by the study.

Three adults participated in the study (*n* = 3). Following assessment, they were randomly assigned to a baseline period of either 3, 4, or 5 weeks of self-monitoring of target OCD symptoms, while symptomatology had to remain stable before beginning treatment. More details about the participants and their treatment are found in the Section “Results.”

### Procedure

A telephone interview was conducted before the first appointment in order to assess whether or not candidate’s symptoms were consistent with OCD and whether the current primary obsession was contamination. Prospective participants took part in a semi-structured diagnostic interview in order to establish the presence of a diagnosis of OCD and to assess for other potential comorbid disorders as defined by the DSM-IV-TR (32). The project was approved by the Ethics Research Boards of UQO and Ottawa University. Every participant was met individually in order to complete the consent form and questionnaires. Afterward, therapy took place during a 12-week period. CBT included daily self-monitoring of obsessions and compulsions for each participant, as well as *in virtuo* exposure during therapy sessions. Two follow-ups were conducted by mail in order to determine if therapeutic gains were maintained 4 and 8 months posttreatment.

### Material

#### Equipment

The experimentation was conducted in a CAVE-like system [i.e., similar to Cruz-Neira et al. (33), but not built by the owners of the trademark] located in the Cyberpsychology Laboratory of the Université du Québec en Outaouais. This immersive system is made up of a 10 ft × 10 ft × 10 ft with stereoscopic images projected on all surfaces: four walls, floor, and ceiling [see Bouchard et al. (34) for a detailed description of the hardware]. The experimenter operated the system from outside the closed cube.

**Figure 1:**
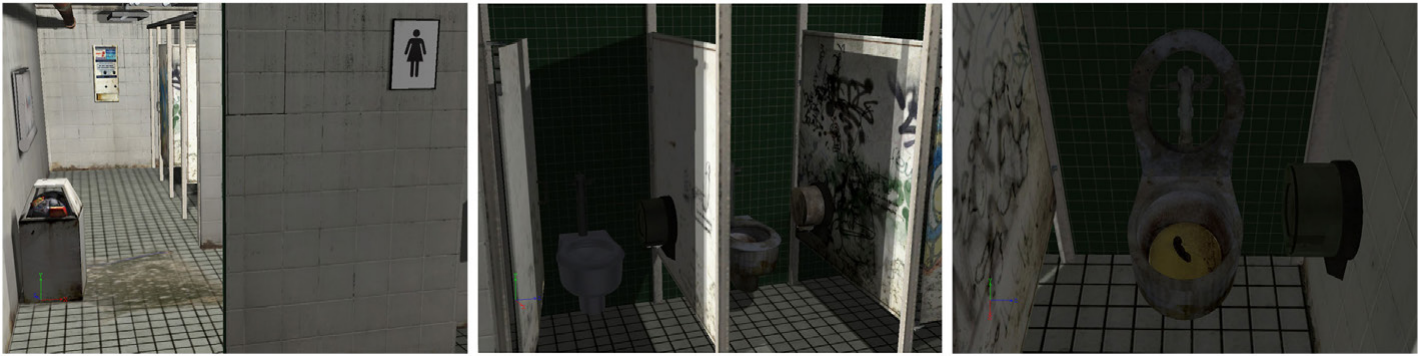
**Screenshots of the virtual environment used for exposure and response prevention in a “contaminated” public toilet**.

Two virtual environments were used, a training environment (“neutral”) and an experimental (“contaminated”) environment. The training environment consisted of an empty room with three windows, a glass door, and a cat resting on a table. The participant could hear the calm sound of a breeze and birds singing. The purpose of this environment was to allow participants to familiarize themselves with the immersion in the CAVE-like system. The training environment was only used once, more specifically at the beginning of the fourth session. The therapeutic environment depicted a public washroom with various degrees of filthiness (see Figure 1) and nothing allowing to eliminate germs (i.e., cleaning products, soap, hand sanitizer).

### Instruments

#### Diagnostic Assessment

The prescreening phone interview was conducted using a questionnaire overviewing different OCD symptoms, including those related to the contamination subtype. Diagnosis was established using the Structured Clinical Interview for DSM Disorders [SCID; (35)]. This semi-structured interview was conducted by two therapists trained in administering the SCID and supervised by a licensed psychologist. The establishment of primary and comorbid diagnosis was based on clarity and causal sequence of the clinical presentation. If there was ambiguity with respect to the primary/secondary diagnosis, a consensus was reached between the interviewers and supervisor.

#### Measuring Therapeutic Progress: Self-Monitoring

The primary measure of treatment efficacy was participants’ self-monitoring of obsessions and compulsions. Participants’ specific impulsive thoughts and compulsive behaviors were operationalized during the first assessment session. Daily self-monitoring based on forms using a similar design (36) were provided to participants who had to rate the presence (i.e., the proportion of the day during which the intrusive thoughts or impulses were present) and intensity (i.e., the strength of intrusive thoughts and compulsive behaviors) of their primary obsession and primary compulsion. These were recorded on a self-rating form that used category partitioning (37) according to the following scale: “none,” 0; “minimal,” 1–20; “few,” 21–40; “average,” 41–60; “a lot,” 61–80, and; “extreme,” 81–100. The literature on OCD suggests that percentages should be preferred over hours since, in some cases, intrusive thoughts or compulsive behaviors are numerous but short in duration, whereas in others, obsessions are less frequent but last longer (38). Participants were informed of the importance of completing the self-monitoring every night in order to increase therapy’s success.

#### Symptom Assessment

In order to provide a more detailed measure of frequency, severity, and diversity of OCD symptoms, therapists administered the YBOCS [(39, 40); French translation by Mollard et al. (41)]. The YBOCS is a 10-item scale administered by therapist in the form of a semi-structured interview to measure the severity of obsessions and compulsions. Each item was rated on a five-point scale ranging from 0 to 4; 0 reflecting no symptoms and 4 reflecting extremely severe symptoms. In order to include participants with fear of contamination as their main subtype, participants had score higher on this subscale compared with other subtypes.

#### Measure of Global Functioning

The Trait-Anxiety subscale of the State-Trait-Anxiety Inventory [TAI-Y2; (42); validated in French by Gauthier and Bouchard (43)] was used to document the general level of anxiety. Daily functioning was assessed with the Evaluation of Actual Life Functioning [EALF; (44)] measuring seven different life domains, namely, (a) occupation or employment, (b) education, (c) social life, (d) hobbies, (e) entertainment, (f) holiday, and (g) everyday activities (cleaning, shopping, etc.). For each life domain, participant had to indicate on a Likert-type scale ranging from 1 (no problem) to 9 (severe difficulties) the extent to which the OCD symptoms had influenced each life domain (1 item per life domain). An average was then calculated.

#### Measures Related to Treatment Using VR

Before the first immersion, participants completed the Immersive Tendencies Questionnaire [ITQ; (45)]. This questionnaire measures participants’ immersive susceptibility in a virtual environment by assessing their immersive tendency when performing other activities (i.e., reading a book, watching a movie). The Simulator Sickness Questionnaire [SSQ; (46); validated in French by Bouchard et al. (47)] was used after each immersion to measure the extent to which participants experienced side effects induced by the immersion in VR (i.e., nausea, eye fatigue, dizziness). Following a description of the CBT, participants were asked to complete the French adaptation by Borkovec and Nau (48) Client Satisfaction Questionnaire (CSQ). This measures participant’s perceived credibility in regard to proposed treatment using 5 items assessed on a scale ranging from 0 to 10.

#### Measure of Therapeutic Alliance

Therapeutic alliance was measured using the French version of the Working Alliance Inventory [WAI; (49, 50)]. The WAI is a self-administered questionnaire, containing 36 items that measures participant’s perspective on three components of the therapeutic alliance between participant and therapist, as defined by Bordin (51). It uses Likert-type rating scales, ranging from 1 (not true at all) to 7 (completely true) with a maximum score of 252.

### Treatment

Cognitive–behavioral therapy unfolded according to a standardized treatment protocol using a guided manual for therapists (29). Individual weekly therapy sessions lasted 60 min. Typically, each session would begin with a discussion on previous week’s homework and self-monitoring of obsessions and compulsions, followed by exposure in VR (i.e., touching walls and toilet bowls with varying degrees of filthiness) and reviewing the exposure session, performing cognitive restructuring of dysfunctional thoughts, and discussing upcoming homework assignments (52). Homework usually included a review of the didactic material viewed in session, the occasional practice of exposure (see below), and self-monitoring (29).

The first three sessions aimed at case conceptualization and introducing treatment planning. During these sessions, therapist gathered information about obsessive fears and rituals and developed an exposure hierarchy of anxiety-provoking situations. Furthermore, the cognitive–behavioral model of OCD and the rationale for exposure and response prevention were discussed. Principles of exposure in VR were also briefly discussed. Sessions 4–11 consisted of exposure and response prevention in VR (*in virtuo* exposure). The first exposure sessions were devoted to mild anxiety-provoking situations, which eventually progressed to situations causing greater distress. It is important to note that participants were systematically asked to refrain from practicing exposure outside the therapist’s office during sessions 4–7 (i.e., no exposure homework). Homework assignments with *in vivo* exposure outside the therapist’s office were assigned from sessions 8–11. Contextual information relevant to contamination (i.e., “You are in the public toilet of a hospital”) was provided verbally before every exposure session. Following *in virtuo* exposure, therapist would draw participant’s attention toward their dysfunctional thoughts. Response prevention consisted of instructing participants to refrain from any compulsive behavior. Session 12 was devoted to relapse prevention. Self-monitoring was used between sessions not only to assess outcome but also to increase awareness of situations triggering urges to ritualize.

#### Therapists

Two therapists (Ph.D. candidates) with prior experience in the treatment of anxiety disorders with CBT and with previous experience in conducting therapy in research protocols administered all therapy sessions. The therapists had more experience with traditional exposure than *in virtuo*, but had already carried out therapy using VR. They received training and hands-on experience in treating OCD using the CBT manual. Continuous supervision by the senior author, a licensed psychologist with 18 years of experience with CBT for anxiety disorders and 9 years of experience in the treatment of anxiety disorders with VR, ensured proper monitoring and standardized and uniform application of treatment.

#### Adherence to Treatment Protocol

Assessing how treatment is delivered is important in clinical trials (53, 54) and following a clear treatment plan, establishing a good therapeutic alliance, explaining treatment rationale, and setting explicit goals are expected to increase the likelihood of treatment adherence among participants. Each therapy session was recorded. Three tapes were randomly selected for each participant (25%), one from the initial third of therapy, one from the middle, and one from the end of therapy. Two trained independent experimenters blind to patient’s status in the study listened to the tapes and rated them. Recordings were assessed with the help of a checklist adapted from the Competency Checklist for Cognitive Therapist (55) and the Cognitive Therapy Checklist of Therapist Competency (56, 57). Results showed that treatment protocol was respected. More specifically, 93.33% of items were *very well* respected by therapists. Only two items were rated as *fairly respected* at times during therapy (proposed exposure exercises not allowing avoidance, therapist adequately reviewed exercises completed at home).

## Results

### Overall Clinical Impressions

**Figure 2:**
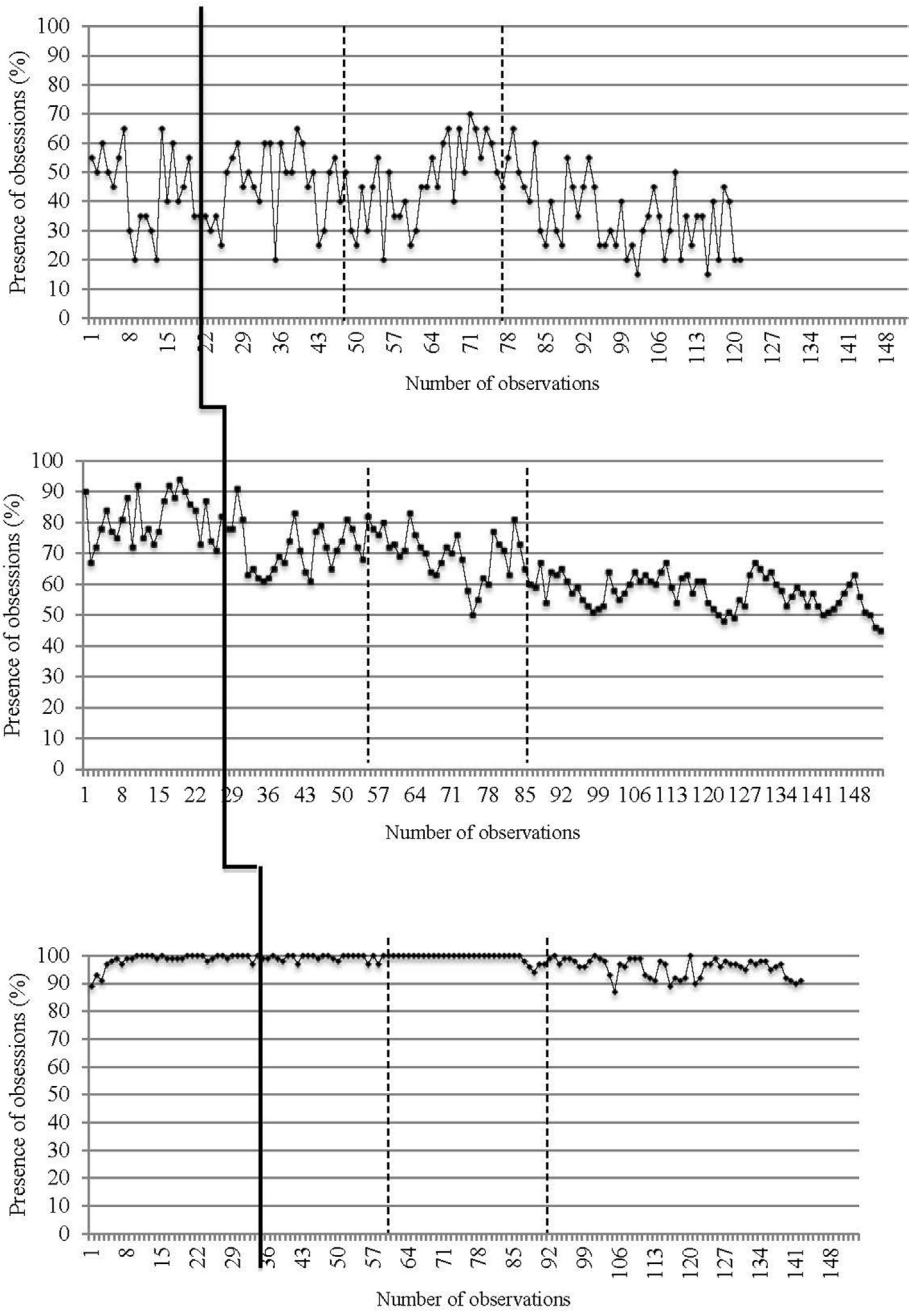
**Presence of obsessions on a daily basis for the three participants**. The first (solid) vertical line represents when CBT was introduced. The second and third (dashed) vertical lines represent when *in virtuo* exposure was conducted.

**Figure 3:**
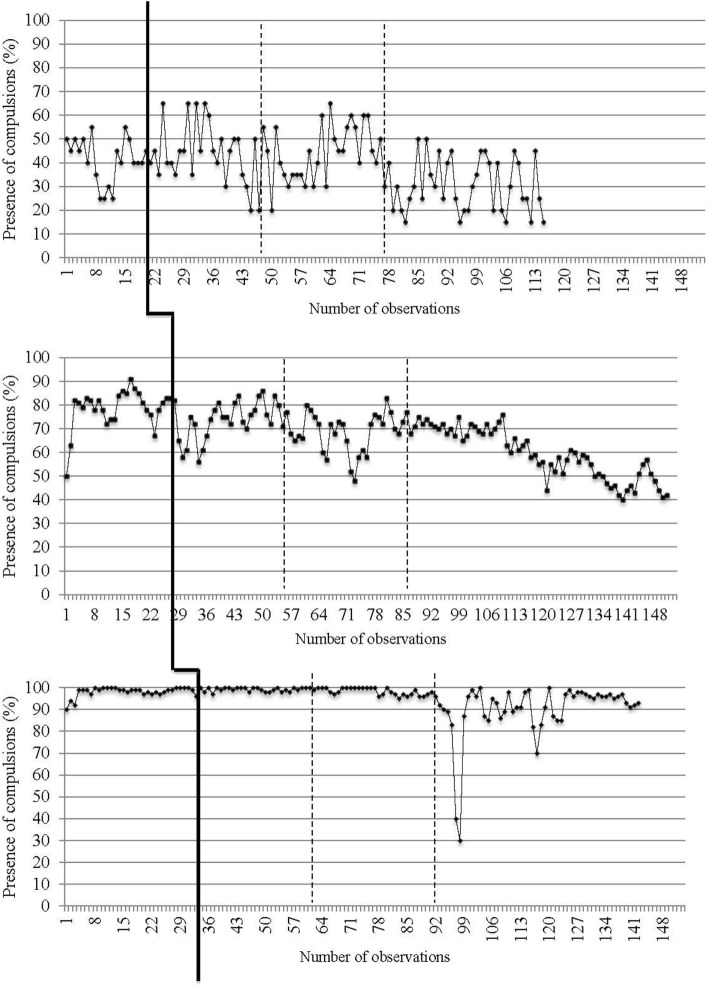
**Presence of compulsions on a daily basis for the three participants**. The first (solid) vertical line represents when CBT was introduced. The second and third (dashed) vertical lines represent when *in virtuo* exposure was conducted.

**Figure 4:**
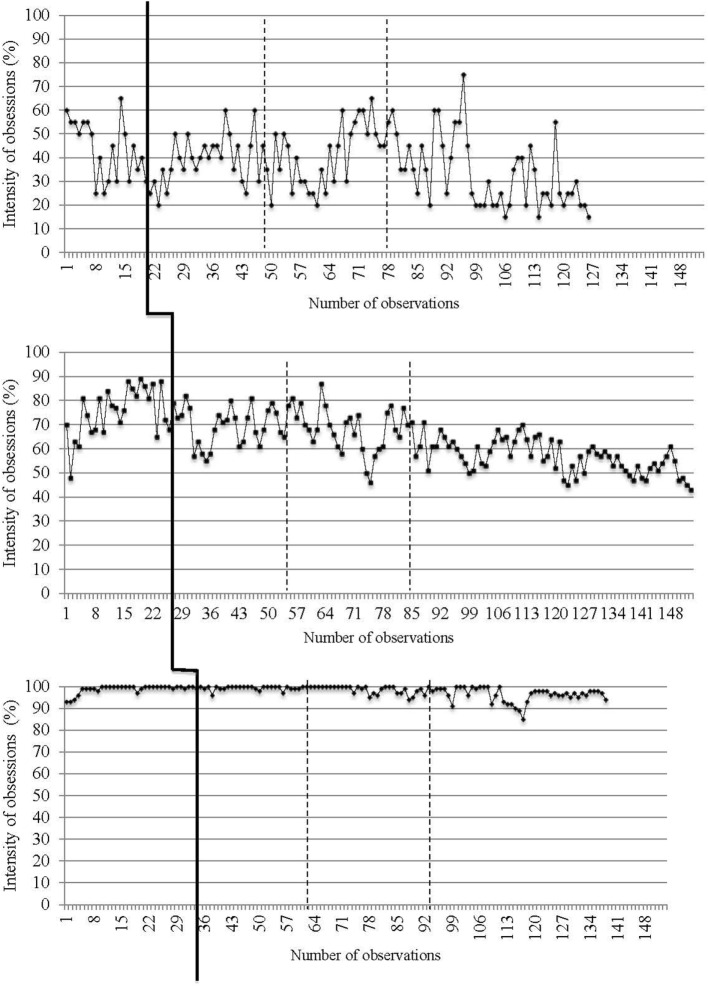
**Intensity of obsessions on a daily basis for the three participants**. The first (solid) vertical line represents when CBT was introduced. The second and third (dashed) vertical lines represent when *in virtuo* exposure was conducted.

**Figure 5:**
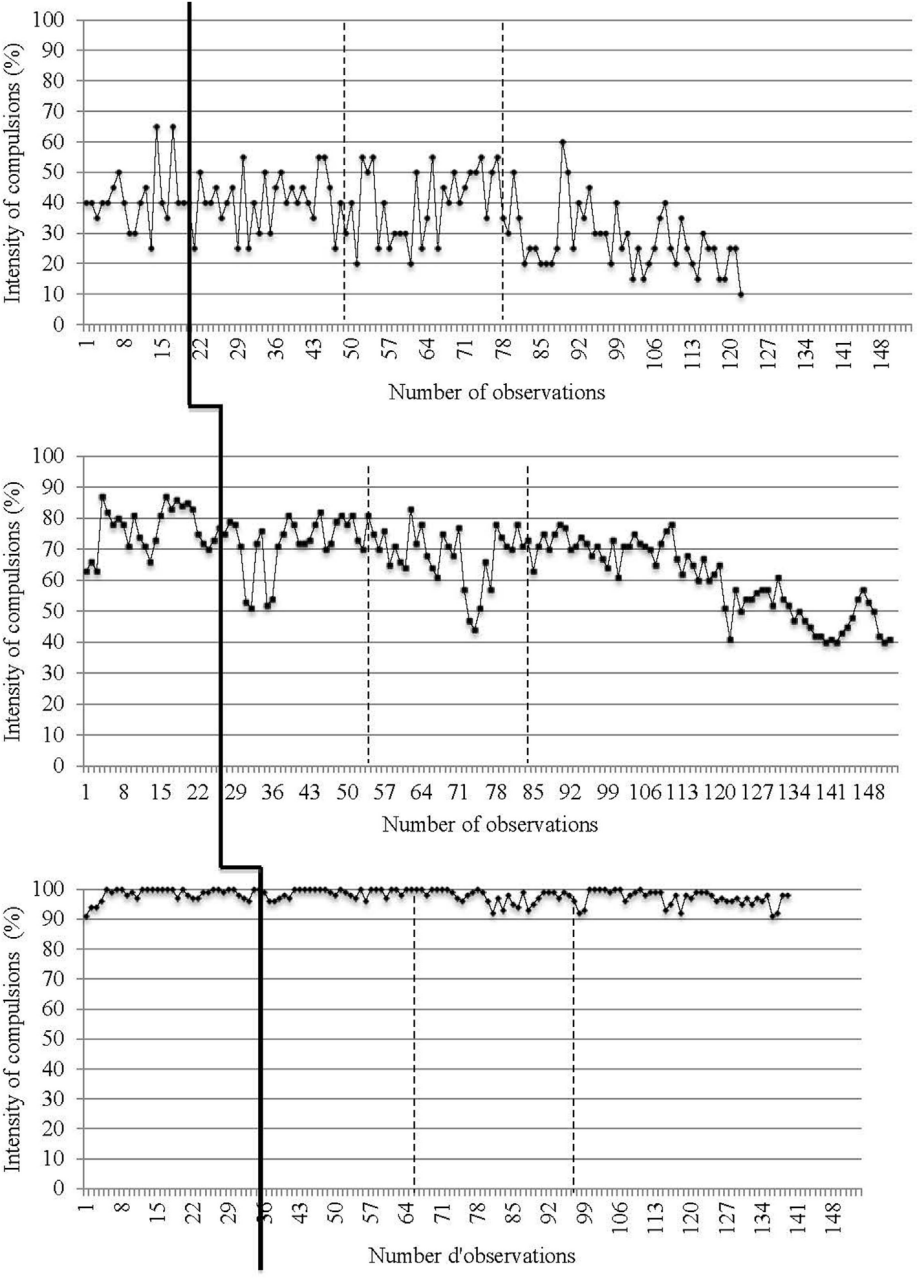
**Intensity of compulsions on a daily basis for the three participants**. The first (solid) vertical line represents when CBT was introduced. The second and third (dashed) vertical lines represent when *in virtuo* exposure was conducted.

Daily data collected from recordings of target OCD symptoms (i.e., presence and intensity of obsessions and compulsions) are reported in Figures 2–5. The moment at which intervention was introduced is indicated with a vertical line.

The first participant is a woman in her mid-20s with a primary diagnosis of OCD with contamination subtype, and a secondary diagnosis of social anxiety disorder, generalized anxiety disorder, and posttraumatic stress disorder. She also displayed symptoms from the symmetry and order subtypes, but they were deemed of a lesser severity than that of contamination. Her Yale–Brown Obsessive–Compulsive Scale [YBOCS; (39, 40)] was in the moderate range (see Table 1). She reported having never received CBT for OCD before. Her main obsessions were related to the contamination of food (cross-contamination) and contamination of her hands and objects in her surroundings. The case conceptualization explored the specific factors contributing to the maintenance of her OCD. They consisted of a difficulty to be aware of direct or subtle avoidance behavior and a tendency to deny her disorder. The eight VR sessions were focused on her daily difficulties (i.e., touching a soap dispenser and the door handle of a public restroom’s toilet stall). Touching the actual floor and walls of the CAVE-like system while immersed in the virtual environment was encouraged in order to increase her sense of presence. Assigned home practice for this participant focused on meal preparation as well as exposure to contaminated objects, while, at the same time, attempting to reduce or avoid rituals.

**Table 1:** **Results for measures of anxiety and daily functioning completed pre- and posttreatments as well as at the fourth and eighth month follow-ups**.

	Participant 1				Participant 2				Participant 3			
Measures	Pre	Post	Follow-up 4 months	Follow-up 8 months	Pre	Post	Follow-up 4 months	Follow-up 8 months	Pre	Post	Follow-up 4 months	Follow-up 8 months
YBOCS	22	14	16	21	31	14	11	11	30	21	23	27
TAI-Y2	32	31	29	28	61	60	46	46	56	59	54	64
EALF	3.5	2.3	2.3	2.3	5.3	3.0	1.3	1.5	7.8	5.5	7.5	8.3

*YBOCS, Yale–Brown Obsessive–Compulsive Scale; TAI-Y2, Trait-Anxiety Inventory (Form Y2 of the STAI); EALF, evaluation of actual life functioning*.

The second participant was a woman in her mid-30s with a primary diagnosis of OCD with contamination subtype and secondary diagnoses of generalized anxiety disorder as well as social anxiety disorder. On the YBOCS, Participant 2 scored within the range of severe OCD symptoms (see Table 1). She had been receiving treatment with a hypnotherapist for about a year. Following a discussion with the hypnotherapist, it was clarified that no therapeutic efforts had been made to treat her OCD and that it was not included in their treatment plan. During treatment, it became clear that her obsessions were mainly triggered by factors such as fear of self-contamination or fear of causing contamination due to “not having washed properly.” Exposure in the virtual environment focused on the theme of contamination within the context of a filthy public restroom described to the patient as “located in a university where students were suffering from flu and cold symptoms.” The participant completed eight VR exposure sessions, followed by cognitive restructuring on exposure experience (i.e., sticky substances, contaminated air, garbage bins). OCDs maintaining factors were also addressed in therapy using cognitive restructuring technique. They included low self-esteem, depressed mood, social isolation, and a tendency to self-criticize. Assigned homework focused on themes addressed during therapy sessions that proved to be difficult to recreate using VR, such as eating without performing compulsions, preparing a meal, and response prevention of compulsions at home, which she identified as a “safe zone” and where compulsions were most significant.

The third participant was a woman in her late 20s with a primary diagnosis of OCD with contamination subtype. This participant also reported obsessive doubts and compulsions of verification, but they appeared to be secondary to the fear of contamination. Her YBOCS score placed her in the severe range of OCD symptoms (see Table 1). She had never received CBT for her OCD. She had also been suffering from a depressed mood in recent weeks. It should be noted that the last session lasted 120 min as sessions 11 and 12 were combined due to time constraints. Her OCD symptoms related to the fear of infecting others, especially through a sexually transmitted infection (i.e., HIV, herpes). The virtual environment depicting filthy public restrooms was presented as “being on a university campus and in a hospital.” Throughout the eight exposure sessions conducted in VR, tasks involved touching unknown sticky surfaces (i.e., floors, walls cabinets, toilet bowls), garbage bins, and a used needle. Following VR exposure, cognitive restructuring was conducted based on discussed topics, such as the possibility of contracting a sexually transmitted infection. Despite having successfully completed VR exposure, Participant 3 showed difficulty with respect to homework (i.e., using public restrooms, preparing dinner for a friend). Maintaining factors were explored and included low self-esteem, difficulty in risk-taking behaviors, high personal standards and the presence of an irritable mood in regard to her general dissatisfaction with her life (employment and relationship).

Traditional visual inspection of graphs was performed for all three participants (see Figures 2–5). Results suggest that interventions had an immediate effect on obsessions and compulsions in the case of Participant 2, as inferred by an apparent change in symptom’s level. For Participant 1 and Participant 3, results are more difficult to interpret on the basis of visual inspection, but suggest that the effect takes place progressively during the course of the treatment. Toward the end of therapy, Participant 1 reported a decrease in OCD symptoms (YBOCS results in Table 1 are in the range of mild symptoms) and the ability to be exposed to anxiety-provoking situations without performing rituals. A decrease in OCD symptoms was also noted for Participant 2. She reported being able to take risks in regard to contamination and to tolerate the associated discomfort. She also mentioned she wanted to keep practicing this technique and apply exposure to objects associated with residual symptoms of OCD. At posttreatment assessment, this participants’ YBOCS score was in the range of mild OCD symptoms; scores remained stable 4 months after treatment (see Table 1) as revealed in follow-up evaluation. As for Participant 3, the candidate reported less avoidance, was able to restructure her unrealistic thoughts and to challenge her fears, as well as give herself better self-appraisal and take greater risks at the end of therapy. Her residual symptoms resided mainly in the practice of *in vivo* behaviors (i.e., using a public restroom); a practice that now generates less anxiety compared with the beginning of treatment. Her posttreatment score on the YBOCS (see Table 1) scaled down to the moderate symptoms range. Scores at follow-ups remained in the same range.

### Time-Series Analyses

To compensate for the subjective nature of visual examination comparing symptom’s during baseline and following the introduction of the intervention, researchers can use more powerful, rigorous, and reliable statistical methods that allow description of events based on mathematical models in order to assess the impact of an intervention (27, 28, 30, 58–62). Time-series analyses [or ARMA (63, 64)] were used as the primary tool to test whether the impact of the CBT using VR was statistically significant. Time-series analyses are statistical procedure that tests the influence of an intervention on series of observations collected at regular intervals, while controlling for autocorrelation in the data. Such analyses allow to test changes in the level and slope of the series (65) after the introduction of the intervention (i.e., compared with baseline self-monitoring of presence and intensity of obsessions and compulsions). The final ARMA intervention models are reported in Table 2.

**Table 2:** **Results for time-series analyses performed on the presence and the intensity of obsessions and compulsions in the three participants**.

Variable participant	Final ARMA model	Jung-box	Intervention parameter (*t*-test)	Significant change occurring at session number
**Presence of obsessions**				
P1	AR-1	19.75	−3.13***	Session 7
P2	AR-1	23.54	−3.42***	Session 1
P3	AR-1	16.63	−4.14***	Session 10
**Presence of compulsions**				
P1	AR-1	25.81	−2.97**	Session 6
P2	AR-1	21.16	−5.80***	Session 9
P3	AR-2	9.55	−4.56***	Session 8
**Intensity of obsessions**				
P1	AR-1	21.49	−4.86***	Session 9
P2	AR-1	23.15	−2.75***	Session 1
P3	AR-1	20.72	−3.53***	Session 9
**Intensity of compulsions**				
P1	AR-2	18.62	−4.28***	Session 8
P2	AR-1	9.50	−7.09***	Session 10
P3	AR-1	9.46	−3.14***	Session 8

*AR, autoregression model; MA, moving average model*.

**p < 0.01, ***p < 0.001.

The ARMA time-series analyses (63, 64) confirmed our hypotheses that the impact of the intervention is statistically significant for all participants. This effect was manifested clearly immediately after the introduction of treatment in the case of presence and intensity of obsessions for Participant 2, and after a few sessions of exposure (between sessions 4 and 11) in regard to other variables and other participants. The magnitude of the impact of the intervention remains greater for Participant 1 and 2 than for Participant 3.

### Self-Reported Measures at Pre- and Posttreatment and Follow-Ups

Table 1 summarizes results in terms of anxiety and general daily functioning of the three participants. These data were not used to demonstrate the effectiveness of the treatment, but rather as descriptive clinical add-on to the ARMA analyses. With results obtained on the YBOCS, it is possible to observe that obsessive and compulsive symptoms showed clinical improvement during the course of treatment for all three participants. Results vary among participants at follow-ups, with Participants 2 showing lasting improvements at follow-ups and Participant 1 showing relapse on the Y-BOCS at the 8-month follow-up. Participant 3 did not show much improvement at the last follow-up.

### Treatment and VR Experience-Related Questionnaires

Participants considered the combined VR with CBT treatment as highly credible (CSQ total score: median = 49.67 on a maximum of 50). The therapeutic alliance established between participants and therapists was strong (WAI total score: median = 237 on a maximum of 252). Participants’ reported a high propensity to be immersed in a virtual environment (ITQ total score: median = 81.67 on a maximum of 126). In terms of unwanted negative side effects experienced after immersion in VR, participants reported low levels of unwanted negative side effects, with slight variations from one session to the next.

## Discussion

The objective of this study was to examine the potential efficacy of using VR to conduct exposure with people suffering from OCD with contamination fears. To this end, the presence and intensity of obsessions and compulsions were documented in a single-case study where patients are used as their own control with treatment introduced at different moments in order to evaluate if its impact follows the introduction of the intervention. After analysis, results provide preliminary evidence for the effectiveness and usefulness of VR in the treatment of OCD.

Based on visual inspection of data and time-series analyses, it is possible to believe the intervention significantly improved OCD symptoms for each participant. The intervention’s effect is clearer in regard to obsessions reported by Participant 2, but this effect seems to appear later in exposure sessions for the other two participants, both for obsessions and compulsions. It is interesting to note that the “contaminated” virtual environment was more akin to Participant 2’s specific obsessions and compulsions (i.e., fear of contact with germs that could then contaminate someone else), compared with Participant 1 (i.e., contamination of food) and Participant 3 (i.e., contracting a sexually transmitted infection).

At the time of assessment, YBOCS score for all participants was contained in the moderate to severe range. Following treatment, Participants 1 and 2 reported a score in the mild range of the scale, thus indicating a clear reduction of obsessive–compulsive symptoms. These gains were maintained up to 4 months (follow-up) after the end of treatment. As for Participant 3, her YBOCS score was reduced from the severe to moderate range following treatment and was maintained until 4-month follow-up. For all three participants, there was a slight loss in therapeutic gains as indicated by results obtained on the YBOCS at 8-month follow-up. As with OCD symptoms, trait anxiety improved for Participant 2, but this improvement was less pronounced for Participant 1 and would have increased at 8-month follow-up for Participant 3. A similar pattern is observed with participants’ daily functioning.

Given these results, we are questioning the duration of the treatment used in this pilot study, as well as whether booster sessions would have been useful in the maintenance of therapeutic gains. It is possible that 12 sessions of CBT, including 8 sessions of *in virtuo* exposure, are insufficient for the treatment of a chronic and complex disorder such as OCD. Indeed, length of CBT can vary between 5 and 22 sessions of CBT (66). In addition, we cannot deny the impact of each participant’s individual factors on treatment. To this effect, a study led by Keijsers et al. (67) highlighted the detrimental impact of variables such as initial severity of OCD, comorbid depression, chronicity of OCD as well as client’s motivation on the prognosis of patients suffering from OCD. In the case of our study, Participant 3 had been suffering from severe OCD for 5 years, showed depressed mood and had difficulty completing exposure outside of the therapist’s office. This reluctance appeared to be caused by her depressed mood, which negatively influenced her motivation, and fear of exposure homework. Fear of exposure was present but easier to manage in VR. Following these considerations, a treatment of longer duration including more VR exposure sessions would have possibly promoted better outcome and encouraged generalization of treatment gains.

In regard to patient’s perception of the treatment, participants positively assessed CBT with *in virtuo* exposure. All three participants exhibited high susceptibility to immersion in a virtual environment and felt little unwanted negative side effects during exposure. A strong therapeutic alliance was created by therapists. Following these considerations, our results support the potential of VR in the treatment of OCD.

This pilot study is not without limitations. Since the study is based on an individual protocol with three participants, generalization of the results is limited. In addition, our sample was composed entirely of women. In this same perspective, differences in symptom’s severity may also blur the results. The duration of the treatment may need to be adjusted and adapted to other CBT protocols [e.g., Ref. (1)]. Given the promising results of this study, it would be important to reproduce the methodology with a larger and more diverse sample (gender, age, and ethnicity) as part of a controlled study protocol with random assignment by comparing *in virtuo* treatment with traditional *in vivo* treatment. It would also be interesting to explore other subtypes of OCD (i.e., symmetry, moral and religion, verification) as well as its impact on chronicity and severity of symptoms. In this regards, VR environments are being developed for other subtypes of OCD [e.g., Ref. (68)].

Participants in this study all reported anecdotes about how they were able to expose themselves to some particular fears in VR with moderate to severe anxiety (i.e., touching the floor of a public restroom), but felt they would not have been able expose themselves to such stimuli *in vivo* at that moment in their treatment. It is possible that VR allows participants to practice exposure technique during session with the assistance of a therapist and help them be better able to implement exposure *in vivo* or as homework.

In conclusion, we wish to emphasize that VR has proven itself in the field of psychological research, but appears in its infancy in regard to its use in clinical practice. Recently, some researchers have focused their attention on the implementation mechanisms of this new technology to understand the reasons for its under-utilization amongst clinicians. A survey of 262 therapists by Schwartzman et al. (69) found that clinicians are reluctant to use VR due to beliefs that using such technology requires training, equipment, and high financial costs. In addition, most therapists reported not knowing the benefits and applications of VR. Another study led by Bertrand and Bouchard (70) demonstrated that the intention to use VR is mostly predicted by the therapists’ perception of its usefulness as a part of treatment. To ensure wider and more efficient use of this technology in clinical practice, the dissemination of knowledge about the various applications and the numerous benefits of VR is essential to correct erroneous beliefs held by clinicians. Schwartzman and colleagues discussed the importance of informing clinicians through conferences and scientific journals, and especially to correct myths about costs. Dissemination of information related to VR and is applications may encourage greater use in clinical settings and consequently improve access of VR for patients.

## Author Contributions

This study is part of the doctoral thesis of ML, Ph.D. Candidate. The second author was the thesis supervisor. All authors listed, have made substantial, direct and intellectual contribution to the work, and approved it for publication.

## Conflict of Interest Statement

The authors declare that the research was conducted in the absence of any commercial or financial relationships that could be construed as a potential conflict of interest.
